# Supplementary material for: Protein-inspired antibiotics active against vancomycin- and daptomycin-resistant bacteria
Source: Nat Commun. 2018 Jan 2;9:22. doi: 10.1038/s41467-017-02123-w (PMC5750218; doi:10.1038/s41467-017-02123-w)
Supplement: Supplementary file 1 — Supplementary Information [file 41467_2017_2123_MOESM1_ESM.pdf]

SUPPLEMENTARY METHODS

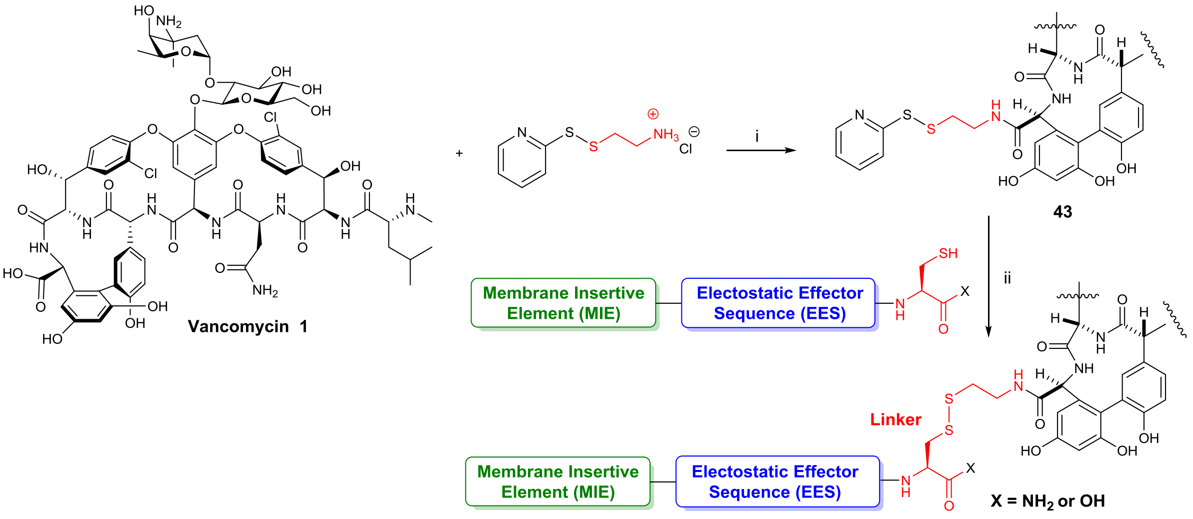

Supplementary Figure 1 | Synthesis of vancomycin derivatives via disulphide ligation.

i) HBTU, HOBt, DIPEA, DMF; ii) DIPEA, H_2O, acetonitrile.

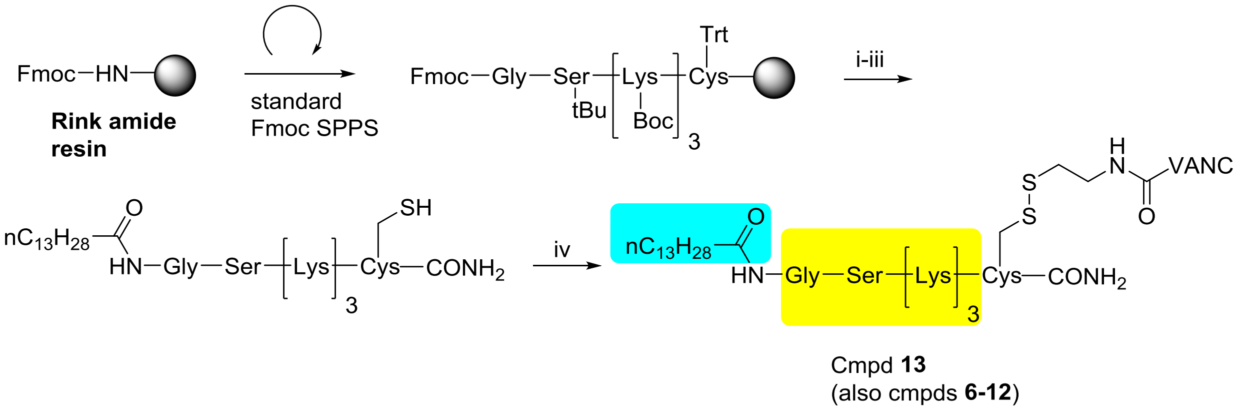

Supplementary Figure 2 | General Synthetic Route to C-terminal amide derivatives obtained via disulfide ligation using general procedures B, E, F (exemplified for compound 13, used to synthesise 6-13).

i) 20% piperidine in DMF; ii) $C_{13}H_{28}CO_2H$, HBTU, DIPEA, DMF; iii) TFA/EDT/TIPS/H_2O (90/5/2.5/2.5 v/v/v/v), rt, 4 h; iv) 2-[(2-Pyridinyl)dithio]ethanamino-vancomycin, DIPEA, ACN/H_2O (1:1), rt, 1 h.

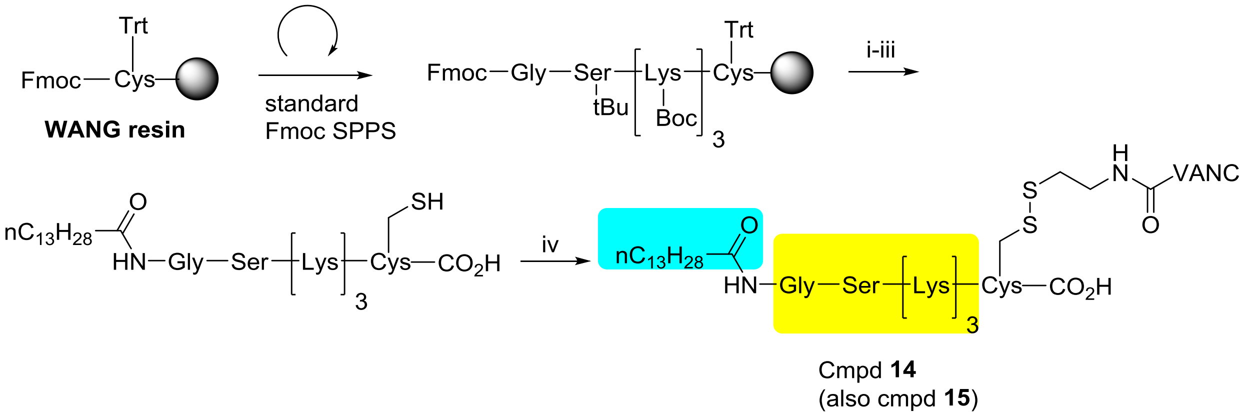

Supplementary Figure 3 | General Synthetic Route to C-terminal acid derivatives obtained via disulphide ligation using general procedures B, E, F (exemplified for compound 14 and used to synthesise 14 & 15).

i) 20% piperidine in DMF; ii) $nC_{13}H_{28}COOH$, HBTU, DIPEA; iii) TFA/EDT/TIPS/H_2O (90/5/2.5/2.5 v/v/v/v), rt, 4 h; iv) 2-[(2-Pyridinyl)dithio]ethanamino-vancomycin, DIPEA, ACN/H_2O (1:1), rt, 1 h.

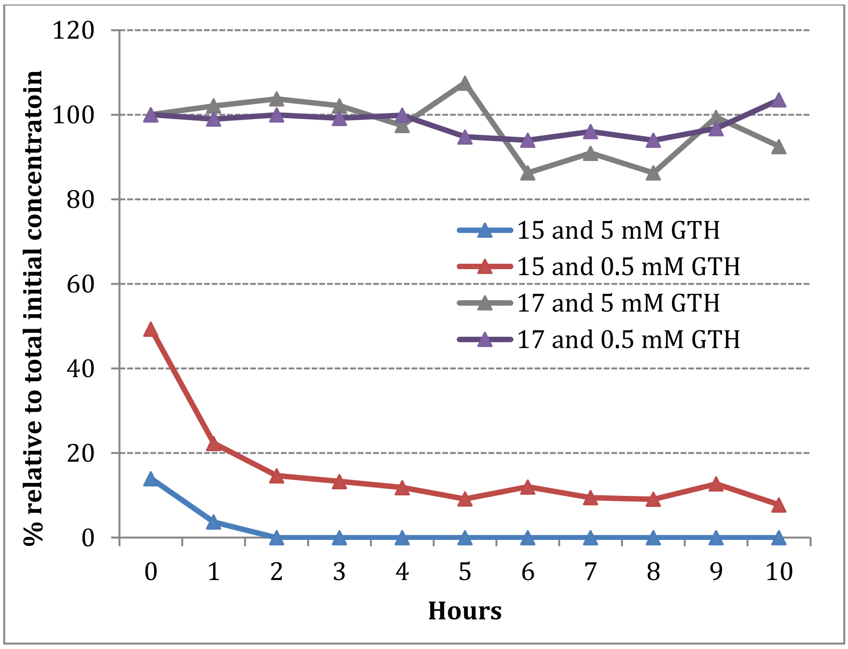

Supplementary Figure 4 | Comparison of stability of 100 µM of S-S linked (Cmpd **15**) and C-linked (Cmpd **17**) analogues in the presence of glutathione (n = 1).

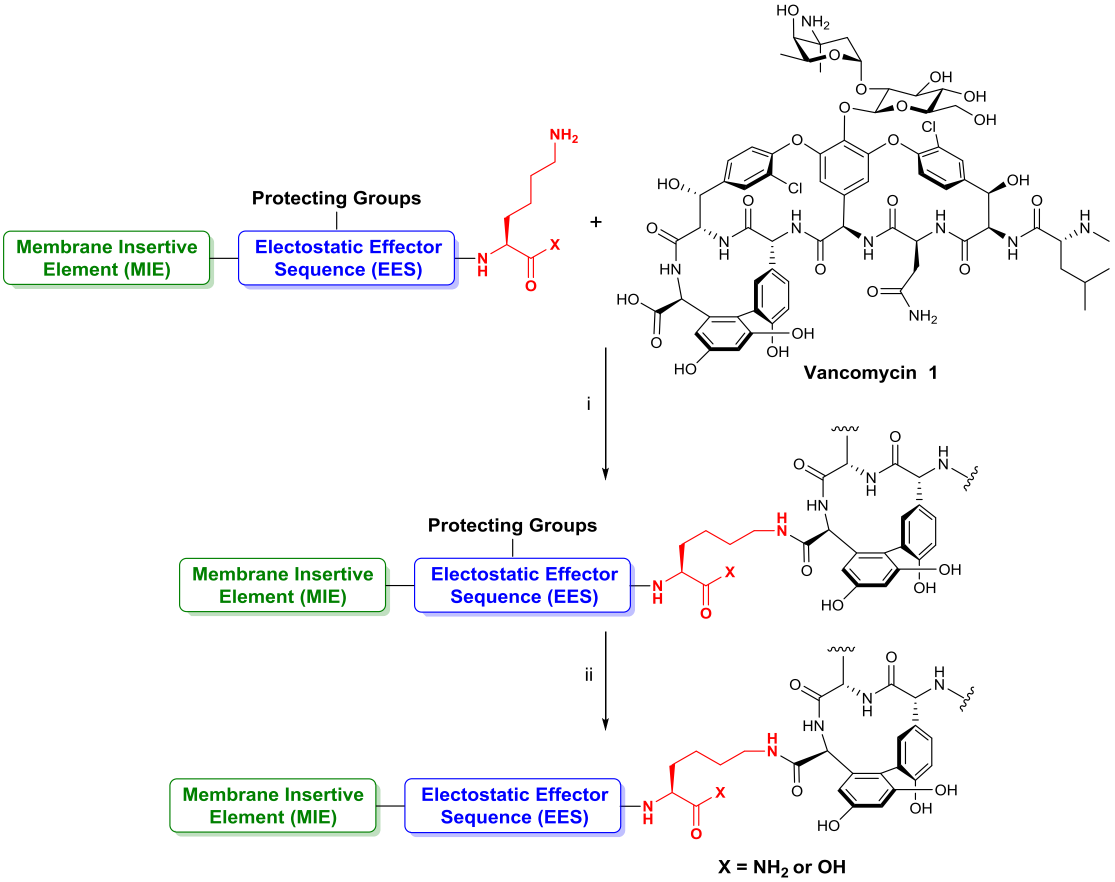

Supplementary Figure 5 | Synthesis of vancomycin derivatives via lysine linker.

i) HBTU, HOBt, DIPEA, DMF or other coupling reagents ii) Mtt or ivDde deprotection.

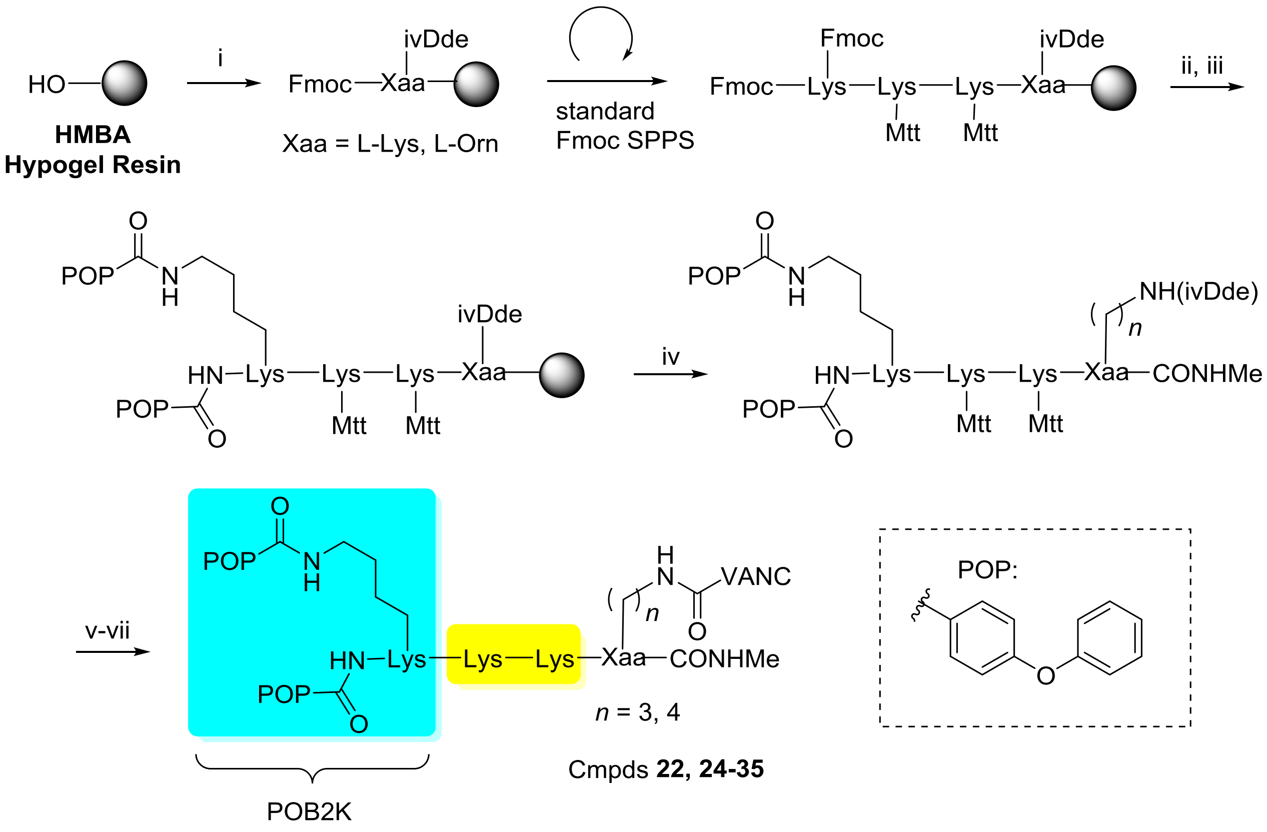

Supplementary Figure 6 | General Synthetic Route to Compounds with Lys-NHMe linker using general procedures B, C, D, E, G (exemplified for compound 24, used to synthesise 22, 24-35).

i) Fmoc-L-Lys(ivDde)-OH, DIC, HOBt, DMAP, DMF; ii) 20% piperidine in DMF; iii) POP-COOH, HBTU, DIPEA, DMF; iv) $MeNH_2$, DIPEA, THF, o/n; v) 2% $H_2NNH_2.H_2O$ in DMF; vi) Vancomycin.HCl, HATU, DIPEA, DMF; vii) 93:5:2 DCM/TES/TFA, 60 min.

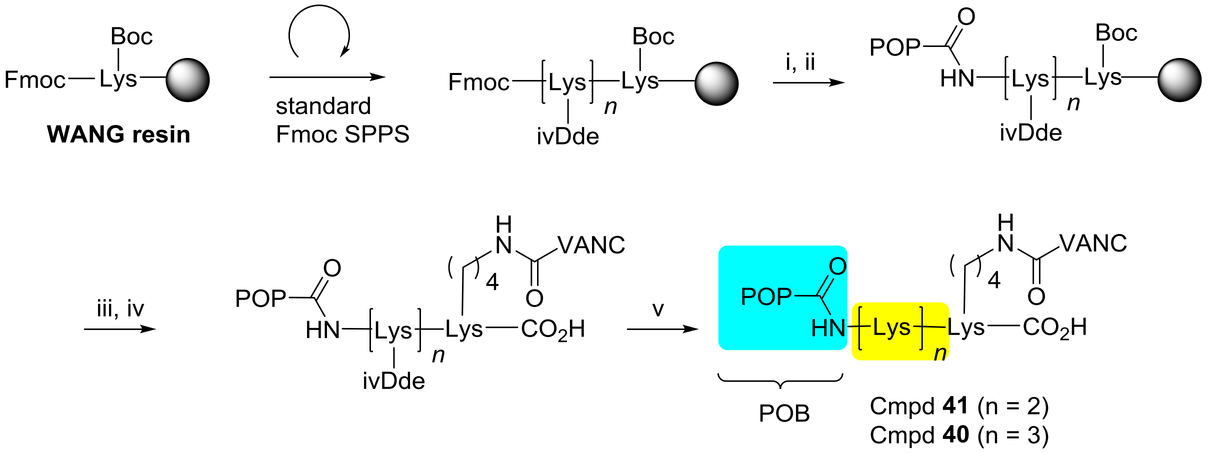

Supplementary Figure 7 | Synthetic Route to Compounds 40 and 41 using general procedures B, C, E, G.

i) 20% piperidine in DMF; ii) POP-COOH, HBTU, DIPEA, DMF; iii) 95:2.5:2.5 TFA/TIS/H_2O; iv) Vancomycin.HCl, HATU, DIPEA, DMF; v) $H_2NNH_2.H_2O$ in DMF.

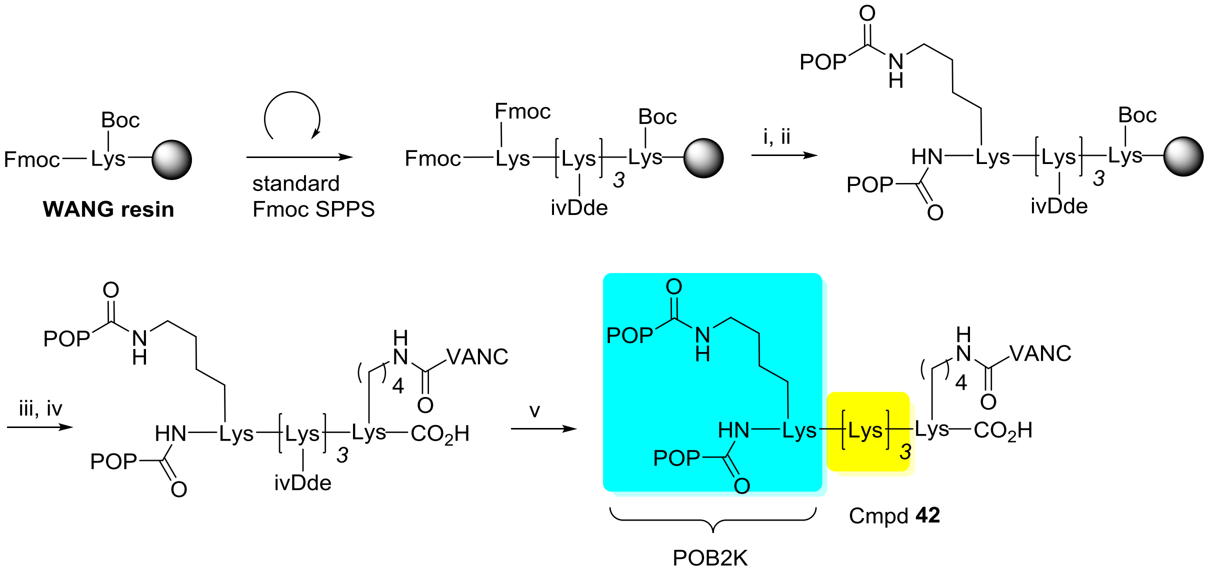

Supplementary Figure 8 | Synthetic Route to Compound 42 using general procedures B, C, E, G.

i) 20% piperidine in DMF; ii) POP-COOH, HBTU, DIPEA, DMF; iii) 95:2.5:2.5 TFA/TIS/H_2O; iv) Vancomycin.HCl, HATU, DIPEA, DMF; v) $H_2NNH_2.H_2O$ in DMF.

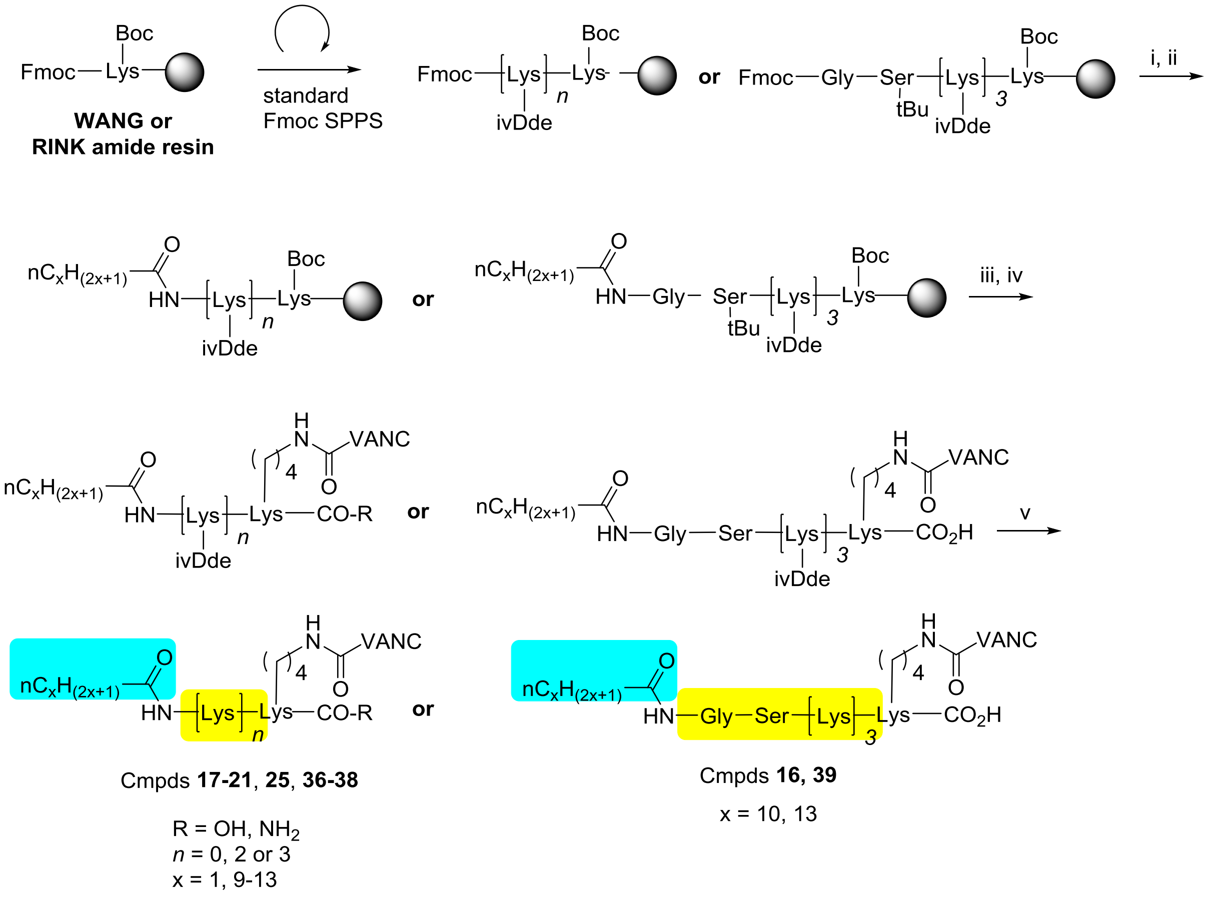

Supplementary Figure 9 | General Synthetic Route to Compounds with Lys-OH or Lys-NH_2 linker using general procedures B, C, E, G (used to synthesise 16-21, 25 and 36-39). i) piperidine/DMF (1:4); ii) (a) nC_xH2_{x+1}-CO_2H, HBTU, DIPEA, DMF or (b) Ac_2O, DIPEA, DMF; iii) 95:2.5:2.5 TFA/TIS/H_2O; iv) Vancomycin.HCl, HATU, DIPEA, DMF; v) 2% H_2NNH_2.H_2O in DMF.

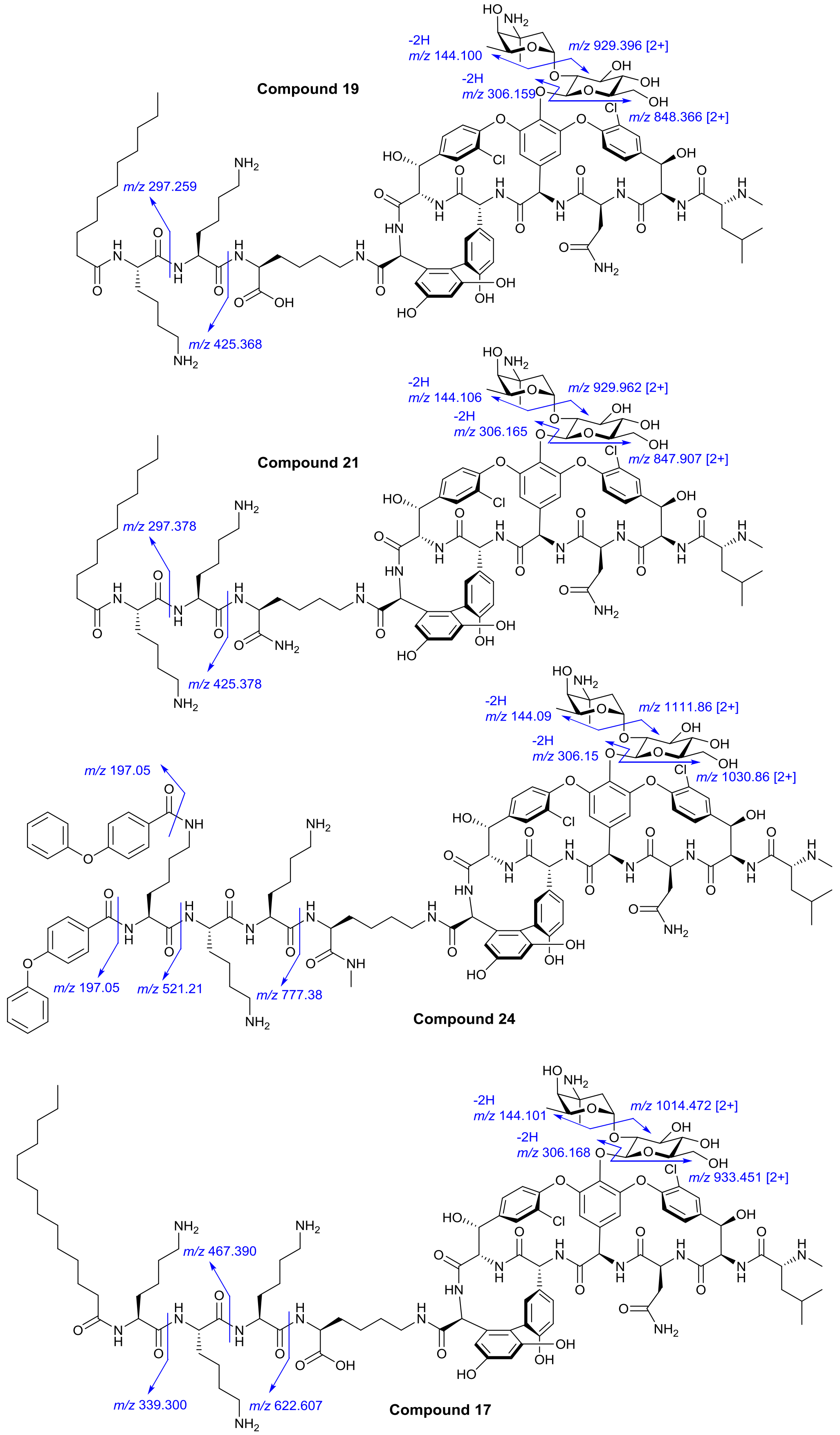

Supplementary Figure 10 | MS/MS assignments of compounds 17, 19, 21, and 24.

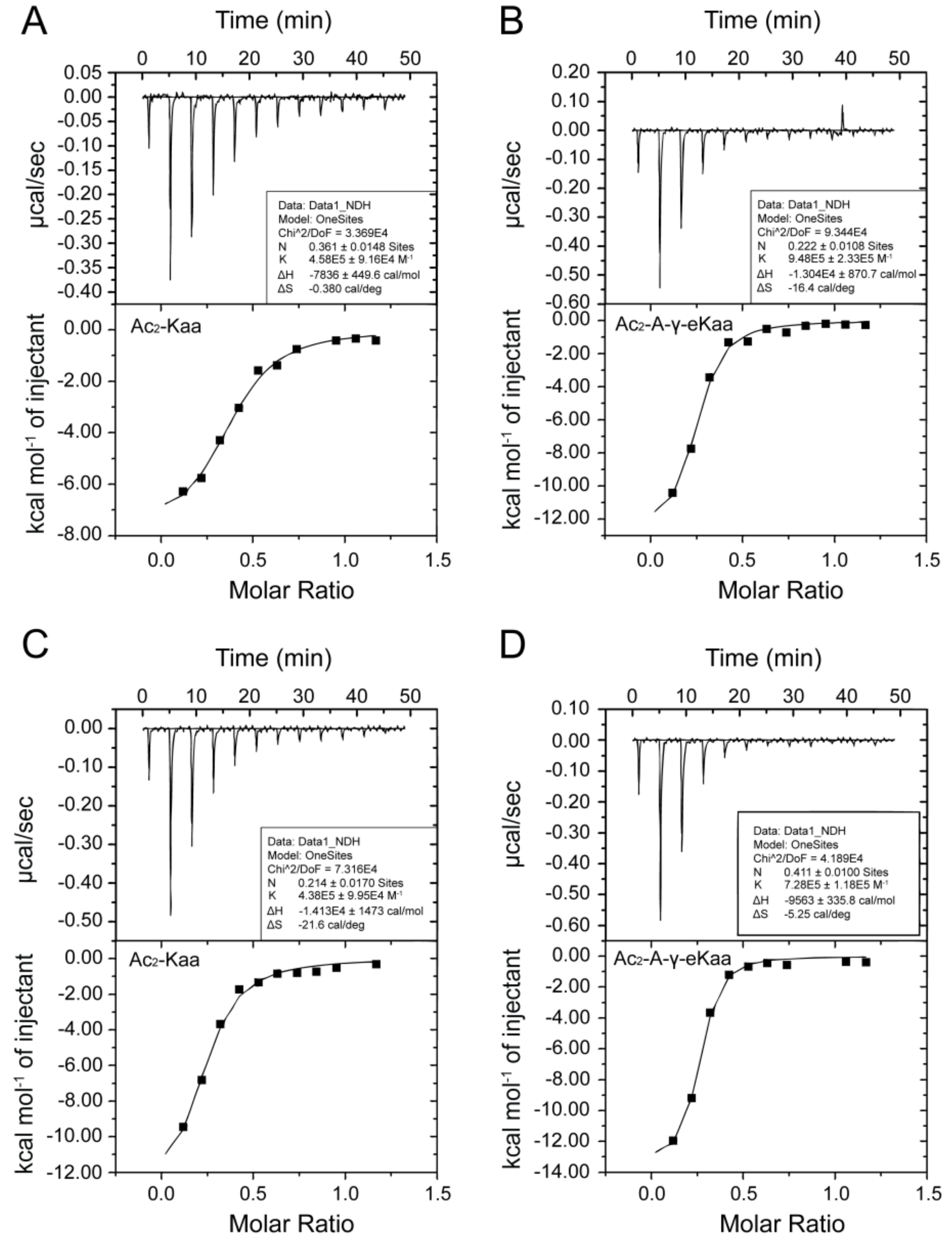

Supplementary Figure 11 | Measurement of Ligand Binding by ITC. Experimental titration curve upon complexation of **17** (A and B) and **24** (C and D) with ligands Ac_2-Kaa and Ac_2-A-*γ*-eKaa at 25 °C in 0.1 M NaOAc, pH 5.0. Concentrations of vancapticins were 40 µM to ensure they existed in solution in the monomeric form (n = 1 shown).

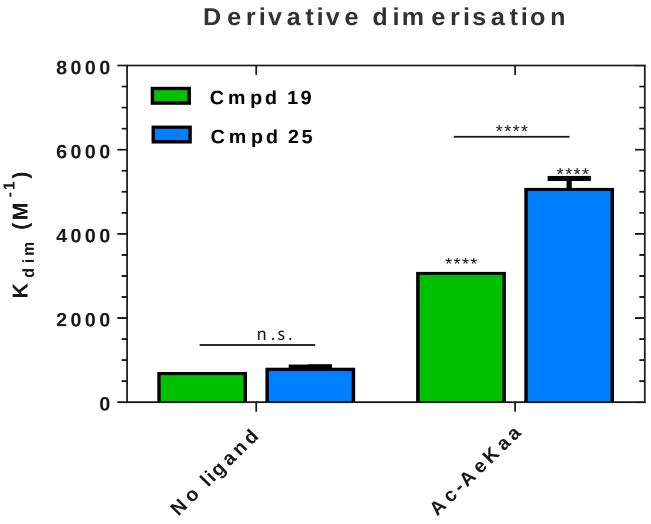

Supplementary Figure 12 | Measurement of Dimerisation by ITC. Mean dimerisation constant (K_{dim}) for dimerisation of vancapticins **19** and **25** in the presence and absence of pentapeptide ligand, Ac-AeKaa. Data derived by ITC at 298K in 0.1 M NaOAc, pH 5.0. Errors are S.D ($n \geq 3$). n.s. = not significant, $P > 0.1$. **** = $P < 10^{-4}$. **25** is an analogue of **19** with the undecanoic acid tail replaced with an acetyl group, used as a control to account for the lipophilic tail which might negatively impact the experiment through potential effects such as micelle formation, aggregation and binding to the walls of the sample cell or plasticware.

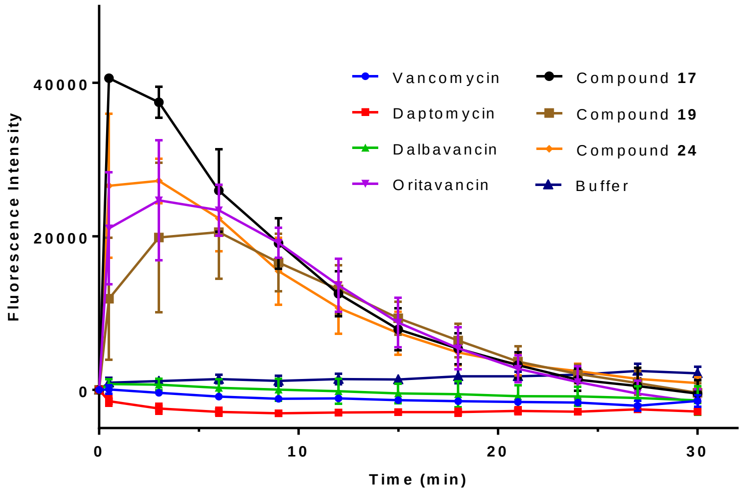

Supplementary Figure 13 | Membrane Depolarisation Assay. Change in fluorescence intensity (665 nm) of the reporter dye diSC3(5) over 30 min following treatment of *S. aureus* MSSA, ATCC 29213 (early exponential phase) with test compounds at 16 µg/mL. Note that the MIC value for oritavancin and compound **17** increases to 1 µg/mL and compound **24** to 2 µg/mL, in polystyrene plates (Supplementary Table 12). Each sample was tested in quadruplicate and independent assays were performed twice showing similar results; results from one assay are presented (n = 4); errors are mean ± S.D.

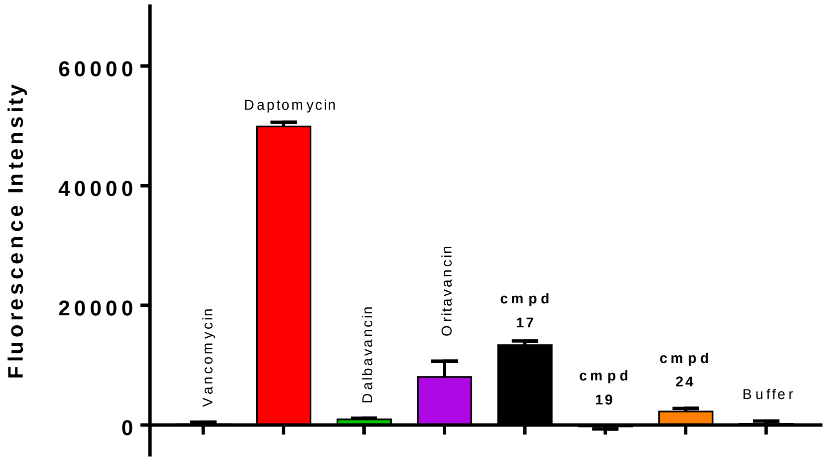

Supplementary Figure 14 | Membrane Permeabilisation Assay. Maximal fluorescence intensity (620 nm) of the reporter dye propidium iodide after 24 min following 1 h incubation of *S. aureus* MSSA, ATCC 29213 (early exponential phase) with test compounds at 16 µg/mL. Note that the MIC value for oritavancin and compound **17** increases to 1 µg/mL and compound **24** to 2 µg/mL, in polystyrene plates (Supplementary Table 12). Each sample was tested in quadruplicate and independent assays were performed twice showing similar results; results from one assay are presented (n = 4); errors are mean ± S.D.

Supplementary Table 1. NMR Assignments. ^{1}H (600 MHz) and ^{13}C (125 MHz) NMR assignments of the acetate salt of compound **19** recorded in DMSO-*d6* compared to vancomycin.

		Vancomycin		Compound 19	
**Subunit**[a]	**Assign-ment**[b]	**^{13}C δ**	**^{1}H δ (mult, *J* Hz)**	**^{13}C δ**	**^{1}H δ (mult, *J* Hz)**
**1 (D-Me-Val)**	C1	172.6		174.7	
	X1	61.8	3.29 (o)	62.1	3.04 (t, 6.6)
	W1	-	n/o	-	n/o
	1a	40.0	1.54 (m), 1.47 (m)	41.2	1.47 (m), 1.39 (m)
	1b	24.0	1.69 (tqq, 6.6, 6.6, 6.6)	24.2	1.71 (m)
	1c	22.8	0.89 (d, 6.6)	23.0	0.88 (d, 6.4)
	1d	22.6	0.85 (d, 6.6)	22.4	0.84 (d, 6.4)
	1e	33.2	2.39 (br s)	33.9	2.29 (br s)
**2 (β-OH Cl Tyr)**	C2	167.0		167.5	
	X2	58.4	4.88 (br s)	58.1	4.88 (br s)
	W2	-	n/o	-	7.80 (v br s)
	Z2	71.1	5.16 (br s)	71.1	5.13 (br s)
	Z2-OH	-	5.83 (br s)	-	n/o
	2a	138.9		139.8	
	2b	127.3	7.38 (v br s)	128.7	7.49 (o)
	2c	126.2		127.2	
	2d	149.8		150.0	
	2e	124.3	7.24 (d, 8.5)	124.3	7.21 (d, 8.5)
	2f	127.3	7.52 (d, 8.5)	127.3	7.49 (d, 8.5)
**3 (Asp)**	C3	171.1		170.0[c]	
	X3	50.9	4.32 (v br s)	50.9	4.40 (v br s)
	W3	-	6.62 (v br s)	-	n/o
	3a	37.3	2.44 (o), 2.13 (br d, 13.5)	37.8	2.35 (m), 2.14 (m)
	C3'	170.7		170.1[c]	
	C3'-NH_2		7.37 (br s), 6.93 (br s)		7.67 (br s), 6.83 (br s)
**4 (Tyr)**	C4	169.5		169.8	
	X4	54.9	5.74 (d, 8.2)	54.9	5.73 (d, 7.7)
	W4	-	n/o	-	8.23 (v br s)
	4a	134.6		134.2	
	4b	107.1	5.54 (br s)	107.0	5.51 (s)
	4c	152.2		152.1	
	4d	131.9		132.0	
	4e	151.3		151.4	
	4f	104.6	5.19 (s)	104.6	5.21 (s)
	C5	169.1		169.3[c]	
**5 (HPG)**	X5	53.7	4.42 (d, 5.7)	53.8	4.43 (br s)
	W5	-	8.63 (br s)	-	8.55 (br s)
	5a	126.2		126.2	
	5b	135.6	7.16 (br s)	135.7	7.22 (s)
	5c	121.6		122.1	

		Vancomycin		Compound 19	
	5d	155.1		155.3	
	5d-OH	-	n/o	-	n/o
	5e	116.2	6.71 (d, 8.5)	116.3	6.68 (d, 8.5)
	5f	125.5	6.76 (dd, 8.2, 1.8)	125.2	6.73 (br d, 8.5)
**6 (β-OH Cl Tyr)**	C6	167.8		168.9[c]	
	X6	61.8	4.18 (d, 11.4)	62.4	4.21 (v br d, 11.0)
	W6	-	6.68 (br d, 11.5)	-	6.79 (br d, 10.8)
	Z6	71.6	5.10 (br s)	70.7	5.31 (s)
	Z6-OH	-	5.92 (d, 6.3)	-	n/o
	6a	142.5		142.8	
	6b	127.3	7.85 (s)	127.3	7.85 (s)
	6c	127.2		126.0	
	6d	148.3		148.2	
	6e	123.4	7.33 (d, 8.2)	123.2	7.27 (o)
	6f	127.2	7.46 (d, 8.2)	127.6	7.48 (d, 8.2)
**7 (DPG)**	C7	172.6		170.9[c]	
	X7	56.7	4.42 (d, 5.6)	58.1	4.32 (d, 5.1)
	W7	-	8.48 (bd d, 4.0)	-	8.51 (br s)
	7a	136.1		137.5	
	7b	118.0		117.8	
	7c	156.4		156.3	
	7c-OH		n/o	-	n/o
	7d	102.3	6.40 (d, 2.0)	102.2	6.30 (s)
	7e	157.2		157.5	
	7e-OH		9.43 (s)	-	n/o
	7f	105.7	6.24 (d, 2.0)	106.5	6.23 (s)
**Glucose**	G1	101.3	5.25 (d, 7.7)	101.2	5.28 (d, 7.7)
	G2	78.1	3.53 (dd, 8.4, 8.4)	77.8	3.54 (dd, 8.5, 8.5)
	G3	77.0	3.44 (ddd, 8.4, 8.4, 8.4)	77.1	3.43 (dd, 8.4, 8.4)
	G3-OH		5.36 (d, 5.4)	-	n/o
	G4	70.1	3.27 (o)	70.3	3.24 (o)
	G4-OH		5.10 (br s)	-	n/o
	G5	76.7	3.27 (o)	76.7	3.25 (o)
	G6	61.2	3.67 (dd, 11.1, 3.6), 3.57 (o)	61.2	3.67 (d, 10.1), 3.51 (br dd, 10.1, 3.6)
	G6-OH		4.03 (t, 5.2)		n/o
**Vancosamine**	V1	96.7	5.23 (d, 3.4)	97.4	5.21 (s)
	V2	33.2	1.88 (br d, 12.0), 1.75 (br d, 12.0)	35.9	1.74 (o), 1.56 (o)
	V3	53.9		51.4	
	V3-NH_2		n/o	-	n/o
	V4	70.7	3.17 (br s)	72.7	2.98 (br s)
	V4-OH		5.44 (v br s)	-	n/o
	V5	63.1	4.67 (q, 6.4)	63.5	4.65 (q, 6.3)
	V6	16.8	1.06 (d, 6.4)	17.2	1.06 (d, 6.1)
	V7	22.2	1.30 (s)	24.6	1.15 (br s)
**ε-Lys**	K1			174.5	

		Vancomycin		Compound 19	
	K2			53.8	3.85 (dt, 6.2, 6.2)
	K2-NH			-	7.29 (o)
	K3			31.9[c]	1.50 (o)
	K4			21.6	1.39 (o), 1.25 (o)
	K5			28.6	1.52, 1.43 (o)
	K6			38.2	3.20 (m), 3.12 (m)
	K6-NH			-	n/o
**Lys-1**	**Lys-1**				
	K1			170.0	
	K2			53.1	4.07 (dt, 4.5, 8.3)
	K2-NH			-	8.43 (d, 6.8)
	K3			31.0	1.72 (o), 1.52 (o)
	K4			22.3	1.43 (o)
	K5			28.8	1.43 (o)
	K6			39.5	2.62 (t, 6.9)
	K6-NH_2			-	n/o
**Lys-2**	K1			171.9	
	K2			52.5	4.27 (dt, 6.2, 6.2)
	K2-NH			-	7.99 (d, 7.7)
	K3			32.0[c]	1.71 (o), 1.45 (o)
	K4			22.6	1.43 (o)
	K5			27.8	1.55 (o), 1.46 (o)
	K6			38.9	2.73 (m)
	K6-NH_2			-	n/o
***n*-C11**	C1			172.0	
	C2			35.0	2.08 (dd, 6.9, 14.0), 2.05 (dd, 6.9, 14.0)
	C3			25.3	1.44
	C4			28.7	1.22 (o)
	C5			28.7	1.22 (o)
	C6			28.8	1.22 (o)
	C7			29.0	1.22 (o)
	C8			29.0	1.22 (o)
	C9			31.3	1.22 (o)
	C10			22.1	1.26 (o)
	C11			14.0	0.84 (t, 6.4)

[a] Abbreviations: 3,5-dihydroxyphenylglycine (DPG), β-hydroxychlorotyrosine (β-OH Cl Tyr) and 4-hydroxyphenylglycine (HPG).

[b] Vancomycin numbering system and assignments consistent with those previously reported.[1]

[b,c,d] Assignments can be interchanged.

Supplementary Table 2. Plasma Stability. Comparison of compound stability in 50% human plasma (n = 1)

Structure				% compound remaining after incubation in 50% human plasma				
Id	MIE[a]	EEPS[b]	Linker	0h	1h	3h	6h	24h
**1**		vancomycin		100	100	87	91	63
**Cys-based Disulfide Linker**								
**14**	nC13CO[c]	GSKKK	C-OH	100	119	nd	82	18
**15**	nC13CO[c]	KKK	C-OH	100	77	64	51	3
**Lys-based Linker**								
**17**	nC13CO[c]	KKK	K-OH	100	79	79	62	66
**18**	nC10CO[c]	KKK	K-OH	100	87	87	83	93
**20**	nC10CO[c]	-	K-OH	100	101	101	100	89
**23**	POB2K[d]	KK	K-OH	100	107	nd	103	99
**24**	POB2K[d]	KK	K-NHMe	100	100	100	106	89

[a]Membrane insertive element. [b]Electrostatic effector peptide sequence. . [c]nCxCO = n-alkanoyl. [d]POB2K = *N*,*N*'-bis(4-phenoxybenzoyl)-Lys.

Supplementary Table 3. Microsomal Stability. Metabolic stability parameters based on NADPH-dependent degradation profiles in human and mouse liver microsomes (n = 1).

Compound	Species	Degradation half-life (min)	*in vitro* Clint[a] (µl/min/mg protein)	Microsome-Predicted EH[b]
Cmpd **18**	Human	>250	<7	<0.23
	Mouse	236	7	0.24
Cmpd **19**	Human	>250	<7	<0.23
	Mouse	>250	<7	<0.23

[a]Intrinsic clearance. [b]Hepatic extraction ratio

Supplementary Table 4. Cytotoxicity. CC_{50} (μM) after 24h as measured by MTT assay in HepG2 and HEK293 cells grown in the presence of 1% FBS (replicate n = 2).

**Structure**				**Cytotoxicity CC_{50} (μM)**	
Id	MIE[a]	EEPS[b]	Linker	HepG2[c]	HEK293[d]
**1**		vancomycin		6300	5900
**2**		telavancin		>100	>100
**3**		dalbavancin		259	73
**4**		daptomycin		>500	>500
**5**		oritavancin		25	29
**Cys-based Disulfide Linker**					
**6**	nC9CO[e]	GSSKSPS(K)$_6$PGD	C-NH_2	>100	>100
**7**	nC11CO[e]	GSSKSPS(K)$_6$PGD	C-NH_2	65	89
**8**	nC13CO[e]	GSSKSPS(K)$_6$PGD	C-NH_2	10	22
**9**	nC15CO[e]	GSSKSPS(K)$_6$PGD	C-NH_2	4	7
**10**	4-Ph-Bz[f]	GSSKSPS(K)$_6$PGD	C-NH_2	nd	nd
**11**	nC13CO[e]	GSSKSPSKKKPGD	C-NH_2	9	18
**12**	nC13CO[e]	GSSKSPSKKKP	C-NH_2	26	41
**13**	nC13CO[e]	GSKKK	C-NH_2	15	46
**14**	nC13CO[e]	GSKKK	C-OH	4	11
**15**	nC13CO[e]	KKK	C-OH	11	18
**Lys-based Linker**					
**16**	nC13CO[e]	GSKKK	K-OH	18	53
**17**	nC13CO[e]	KKK	K-OH	15	19
**18**	nC10CO[e]	KKK	K-OH	106	108
**19**	nC10CO[e]	KK	K-OH	290	355
**20**	nC10CO[e]	-	K-OH	>500	>500
**21**	nC10CO[e]	KK	K-NH_2	131	113
**22**	nC10CO[e]	KK	K-NHMe	80	47
**23**	POB2K[g]	KK	K-OH	41	47
**24**	POB2K[g]	KK	K-NHMe	100	140
**25**	Ac[h]	KK	K-OH	>500	>500

[a]Membrane insertive element. [b]Electrostatic effector peptide sequence. [c]liver hepatocellular cells. [d]human embryonic kidney cells. [e]nCxCO = n-alkanoyl. [f]4-Ph-Bz = 4-phenylbenzoyl. [g]POB2K = *N*,*N*'-bis(4-phenoxybenzoyl)-Lys. [h]Ac = acetyl.

Supplementary Table 5. Haemolysis. Haemolysis levels of vancapticins and control antibiotics vancomycin **1** and daptomycin **4** against human whole blood at concentrations ranging from 25 to 1600 µg/mL were normalised by subtraction of the signal derived from the running buffer (0.9% NaCl solution) and then compared with a completely hydrolysed sample, in which the human whole blood was incubated with sdH_2O (n = 1).

		**% Human Whole Blood Haemolysis**						
	**Compound**	25 µg mL^{-1}	50 µg mL^{-1}	100 µg mL^{-1}	200 µg mL^{-1}	400 µg mL^{-1}	800 µg mL^{-1}	1600 µg mL^{-1}
**1**	vancomycin	0.1	0.4	0.3	0.8	0.8	0.8	1.5
**4**	daptomycin (with Ca2+)	0	0.4	0	0.3	0.9	0.3	1.6
**14**	nC13CO-GSKKKC(SEtNH-Vanc)-OH			0.4				6.4
**17**	nC13CO-KKK-K(Vanc)-OH	0.15	0.75	0.65	0.85	0.5	1	1.55
**18**	nC10CO-KKK-K(Vanc)-OH			1.4				1.2
**19**	nC10CO-KK-K(Vanc)-OH			0.7				0.5
**22**	nC10CO-KK-K(Vanc)-NHMe			1.9				2.7
**23**	(4-PhO-PhCO)-K(4-PhO-PhCO)-KK-K(Vanc)-OH			-0.6				-0.6
**24**	[(4-PhO-PhCO)-K(4-PhO-PhCO)]-KK-K(Vanc)-NHMe	0.3	0.65	0.25	0.25	0.05	0.2	2.9

Supplementary Table 6. Broth microdilution Minimum Inhibitory Concentration (MIC, μg mL^{-1}) determined with/without addition of Survanta (n = ≥ 2).

	S. aureus ATCC 43300 MRSA[a]			*S. pneumoniae* ATCC 33400			*S. pneumoniae* ATCC 700677 MDR[b]		
**added Survanta** / **Compound ID**	**none**	**1%**	**5%**	**none**	**1%**	**5%**	**none**	**1%**	**5%**
vancomycin **1**	0.5	0.5	0.5	0.5	0.5	0.5	0.5	0.5	0.5
telavancin **2**	0.125	0.1256	0.25-0.5	0.125-0.25	0.25	0.5	0.06-0.125	0.06-0.125	0.25
dalbavancin **3**	0.03-0.06	0.06	0.125	0.06	0.06	0.125	0.06	0.06	0.125-0.25
daptomycin **4**	2	>8	>8	2-4	>8	>8	4	>8	>8
**19**	0.015-0.06	0.015-0.03	0.03-0.06	0.06	0.06	0.125	0.06	0.03-0.06	0.06-0.125
**21**	0.015	0.007	0.03	0.03	0.015-0.03	0.06-0.125	0.015	0.007-0.015	0.06-0.125
**24**	≤0.003	0.007	0.03-0.06	≤0.003	0.06	0.5	≤0.003	0.007-0.06	0.25

[a]MRSA, methicillin resistant *S. aureus*; [b]MDR, multidrug-resistant. Plate type NBS, non-binding surface plate (Corning Cat No. 3641). All plates are 96-well flat bottom. Assays for daptomycin contain 50 μg/mL $CaCl_2$.

Supplementary Table 7. Resistance Frequency. Innate resistance rate determination of *S. aureus* (MRSA) ATCC 43300 (n = 2).

Fold MIC	Inoculum Dilution tube				Average cfu/plate	Resistance frequency
	**Neat** in 100µl		$\mathbf{10^{-1}}$ in 100µl			
**Vancomycin (agarose MIC = 1 µg mL^{-1})**						
8x	0	0	0	0	-	-
4x	3	1	0	0	-	-
2x	130	74	6	0	88	8.33×10^{-10}
1x	3070	>	220	254	2603.33	2.46×10^{-8}
**Compound 19 (agarose MIC = 0.06 µg mL^{-1})**						
16x	40	80	0	0	60	5.68×10^{-10}
8x	108	91	10	0	99.67	9.44×10^{-10}
4x	>	>	295	264	2795	2.65×10^{-8}
2x	>	>	>	>	nd	nd

> = too many colonies to read

Supplementary Table 8. Mouse Pharmacokinetic Individual Mouse Data. Compound plasma concentrations in ng mL^{-1}.

vancomycin IV (2 mg/kg)						vancomycin SC (10 mg/kg)					
Time (h)	M1	M2	M3	Mean IV	SD	Time (h)	M4	M5	M6	Mean SC	SD
0.0833	6650	5570	5150	5790	774	0.250	12500	9710	10000	10737	1534
0.250	4350	3360	3050	3587	679	0.500	10100	9190	9460	9583	467
0.500	2350	1920	1680	1983	339	1.00	3780	4520	7300	5200	1856
1.00	902	767	618	762	142	2.00	1030	1110	1660	1267	343
2.00	197	203	180	193	11.9	4.00	128	96.6	186	137	45.4
4.00	25.8	31.2	25.6	27.5	3.18	8.00	5.46	4.82	8.22	6.17	1.81
8.00	BQL	BQL	BQL	ND	ND	24.0	BQL	BQL	BQL	ND	ND
24.0	BQL	BQL	BQL	ND	ND						
Vancapticin 18 IV						Vancapticin 18 SC (10 mg/kg)					
Time (h)	M1	M2	M3	Mean IV	SD	Time (h)	M4	M5	M6	Mean SC	SD
0.0833	4040	5820	6450	5437	1250	0.250	3700	724	2650	2358	1509
0.250	3030	3950	3720	3567	479	0.500	6830	1030	5490	4450	3037
0.500	1990	2320	2450	2253	237	1.00	9020	829	8820	6223	4672
1.00	1110	1500	1900	1503	395	2.00	4870	297	8380	4516	4053
2.00	325	469	546	447	112	4.00	1370	74.2	2330	1258	1132
4.00	45.4	61.3	102	69.6	29.2	8.00	108	5.48	225	113	110
8.00	BQL	BQL	7.67	ND	ND	24.0	BQL	BQL	BQL	ND	ND
24.0	BQL	BQL	BQL	ND	ND						
Vancapticin 19 IV (2 mg/kg)						Vancapticin 19 SC (10 mg/kg)					
Time (h)	M1	M2	M3	Mean IV	SD	Time (h)	M4	M5	M6	Mean SC	SD
0	BQL	BQL	BQL	ND	ND	0	BQL	BQL	BQL	ND	ND
0.0833	7790	10600	9720	9370	1437	0.250	3570	2440	1940	2650	835
0.250	6160	7670	7080	6970	761	0.500	9120	4020	2250	5130	3567
0.500	5540	6810	5820	6057	667	1.00	12400	7260	6700	8787	3142
1.00	3490	5930	5150	4857	1246	2.00	9990	8880	8620	9163	728
2.00	3070	2320	2540	2643	386	4.00	8770	6170	7180	7373	1311
4.00	1650	1530	1180	1453	244	8.00	2610	2460	2080	2383	273
8.00	197	101	113	137	52.3	24.0	65.5	145	161	124	51.1
24.0	6.95	4.44	BQL	5.70	ND						
Vancapticin 21 IV (2 mg/kg)						Vancapticin 21 SC (10 mg/kg)					
Time (h)	M1	M2	M3	Mean IV	SD	Time (h)	M4	M5	M6	Mean SC	SD
0	BQL	BQL	BQL	ND	ND	0	BQL	BQL	BQL	ND	ND
0.0833	11700	9450	8910	10020	1480	0.250	383	1410	1040	944	520
0.250	3920	4800	8330	5683	2334	0.500	855	2060	1420	1445	603
0.500	5080	4020	3890	4330	653	1.00	1680	4770	2410	2953	1615
1.00	3330	1790	2300	2473	784	2.00	1620	4530	3270	3140	1459
2.00	2280	1320	2230	1943	540	4.00	2630	2800	1520	2317	695
4.00	1110	706	457	758	330	8.00	1450	1380	1270	1367	90.7
8.00	69.8	40.9	37.4	49.4	17.8	24.0	61.0	15.3	58.6	45.0	25.7
24.0	BQL	BQL	BQL	ND	ND						
Vancapticin 24 IV (2.00 mg/kg)						Vancapticin 24 SC (10.0 mg/kg)					
Time (h)	M1	M2	M3[a]	Mean IV	SD	Time (h)	M4	M5[a]	M6[a]	Mean SC	SD
0	BQL	BQL	BQL	ND	ND	0	BQL	BQL	BQL	ND	ND
0.0833	15300	13300	12800	13800	1323	0.250	256	225	199	227	28.5
0.250	13200	8310	8580	10030	2749	0.500	540	597	508	548	45.1
0.500	5520	4530	5640	5230	609	1.00	1450	1710	1050	1403	332
1.00	2630	3350	3050	3010	362	2.00	1410	1610	2740	1920	717
2.00	1140	1410	1080	1210	176	4.00	2030	1850	3160	2347	710
4.00	373	272	209	285	82.7	8.00	1050	710	774	845	181
8.00	102	55.1	88.7	81.9	24.2	24.0	191	320	218	243	68.0
24.0	BQL	3.23	BQL	ND	ND						

Supplementary Table 9. Mouse Pharmacokinetic Parameters (10 mg kg^{-1} SC or 2 mg kg^{-1} IV).

PK Parameters	vanco	18	19	21	24
**IV Dosing, 2 mg kg^{-1}**					
C_0 (ng/ml)	7361	6740	10887	14237	16300
$T_{1/2}$ (h)	0.64	0.77	2.22	1.12	2.29
Vd_{ss} (L/kg)	0.39	0.43	0.267	0.31	0.248
Cl (ml/min/kg)	10.4	8.11	1.98	3.13	2.98
T_{last} (h)	4	ND	18.7	8	ND
$AUC_{0\text{-}last}$ (ng.h/ml)	3211	4208	16800	10833	11033
$AUC_{0\text{-}inf}$ (ng.h/ml)	3236	4245	16867	10923	11200
$MRT_{0\text{-}last}$ (h)	0.59	0.9	2.2	1.6	1.24
$MRT_{0\text{-}inf}$ (h)	0.62	0.9	2.26	1.7	1.39
$AUC_{0\text{-}inf}/AUC_{0\text{-}last}$ (%)	101	101	100	101	101
**SC Dosing, 10 mg kg^{-1}**					
C_{max} (ng/ml)	10737	6290	9967	3557	2347
T_{max} (h)	0.25	0.83	1.67	2.33	4
$T_{1/2}$ (h)	0.8	1.1	3.49	3.4	6.97
T_{last} (h)	8	8	24	24	24
$AUC_{0\text{-}last}$ (ng·h/ml)	11402	16093	59893	23130	20000
$AUC_{0\text{-}inf}$ (ng.h/ml)	11409	16277	60540	23367	22567
$MRT_{0\text{-}last}$ (h)	0.98	1.9	5.32	6.0	7.99
$MRT_{0\text{-}inf}$ (h)	0.99	2.0	5.59	6.3	11
$AUC_{0\text{-}inf}/AUC_{0\text{-}last}$ (%)	100	101	101	101	113
Bioavailability (%)[b]	70.5	77	71.8	43	36.3

Supplementary Table 10. Ligand Antagonism. Concentration of added Ac_2Kaa ligand required to neutralise MIC activity of vancomycin and vancapticin glycopeptides (n = 2).

**Compound ID**	**MIC [µg mL^{-1}]**	**Conc. [µg mL^{-1}]**	**Ac_2Kaa [µg mL^{-1}]**	**Molar excess**
Vancomycin **1**	0.25	0.25	6.25	96
**39**	0.06	0.1	400	30,569
**19**	≤0.004	0.1	100	6,604
**40**	0.03	0.1	12.5	916
**14**	≤0.004	0.1	>3200	252,478
**20**	0.03	0.1	12.5	663
**41**	0.06	0.1	1.56	104
**23**	≤0.004	0.1	200	15,102
**17**	≤0.004	0.1	400	29,472
**18**	≤0.004	0.1	100	7,249
**42**	≤0.004	0.1	1600	131,225

Supplementary Table 11. Dimerisation thermodynamics. Dimerisation thermodynamics of vancomycin and compound **19** in the absence of ligands at 25 °C in 0.1 M NaOAc, pH 5.0

**Antibiotic**	**(M^{-1})**		**(kJ mol^{-1})**	
	**K_{dim}^{a}**	**ΔH_{dim}^{a}**	**$T\Delta S_{dim}$^{a}**	**ΔG_{dim}^{a}**
Vancomycin	750 ± 80	-11.5 ± 0.5	4.9 ± 0.7	-16.4 ± 0.3
Cmpd **19**	1060 ± 140	-13.7 ± 0.1	3.6 ± 0.2	-17.2 ± 0.3

^{a} Data are means ± SD for n=3.

Supplementary Table 12. Broth microdilution Minimum Inhibitory Concentration (MIC, µg mL^{-1}) determined in various plate types and with/without polysorbate-80 against *S. aureus* strains (n = 2-4).

Compound ID	*S. aureus* ATCC 43300 MRSA				*S. aureus* ATCC29213 MSSA			
	**NBS**	**PP**	**PS**	**PS+ P-80**	**NBS**	**PP**	**PS**	**PS+ P-80**
vancomycin **1**	1	2	2	2	1	1	2	1 - 2
dalbavancin **3**	≤0.016	0.063	0.25	≤0.016	≤0.016	0.125	0.25	≤0.016
daptomycin **4**	1	1	1	1	0.5 - 1	1	1	1
oritavancin **5**	≤0.016	1	1	≤0.016	≤0.016	1	1	0.031
**17**	≤0.016	1	1	≤0.016	≤0.016	1 - 2	1	≤0.016
**19**	≤0.016	0.06 - 0.125	0.063	0.063	≤0.016	0.125 - 0.25	0.03 - 0.06	0.031
**24**	≤0.016	1	2	≤0.016	≤0.016	1	2	0.031

MRSA, methicillin resistant *S. aureus*; MSSA, methicillin susceptible *S. aureus*. Plate type/conditions include NBS, non-binding surface plate (Corning Cat No. 3641); PP, polypropylene (Corning Cat No. 3364); PS, polystyrene (Corning Cat No. 3370); PS+P-80, polystyrene with the addition of 0.002% polysorbate-80. All plates are 96-well flat bottom. Assays for daptomycin contain 50 µg/mL $CaCl_2$. MIC measurements are the median of n = 2-4.

Supplementary References

[1]Pearce, C. M. & Williams, D. H. Complete assignment of the ^{13}C NMR spectrum of vancomycin. *J. Chem. Soc. Perkin Trans. 2*, 153–157 (1995)
